# Mainstream and Sidestream Modeling in Oxygen Evolution Electrocatalysis

**DOI:** 10.1021/acs.accounts.5c00439

**Published:** 2025-08-20

**Authors:** Federico Calle-Vallejo

**Affiliations:** † Nano-Bio Spectroscopy Group and European Theoretical Spectroscopy Facility (ETSF), Department of Advanced Materials and Polymers: Physics, Chemistry and Technology, 16402 University of the Basque Country UPV/EHU, Av. Tolosa 72, 20018 San Sebastián, Spain; ‡ IKERBASQUE, Basque Foundation for Science, Plaza de Euskadi 5, 48009 Bilbao, Spain

## Abstract

The oxygen evolution reaction (OER) and oxygen reduction reaction (ORR) are key in numerous electrochemical technologies, such as water electrolyzers, CO_2_ electrolyzers, low-temperature fuel cells, regenerative fuel cells and some metal-air batteries. The OER and ORR tend to be sluggish and catalyzed by scarce and expensive materials, the durability of which is often insufficient. For two decades, computational methods have been regarded as a cost-effective means to explain experimental observations, test hypothesis, and design new materials for these two reactions.

Currently, the most widely used computational model is based on the free energies of the intermediates (*O, *OH, *OOH) and the scaling relations among them. Since the publication of two seminal papers in 2011, the scaling relation between the adsorption energies of *OOH and *OH was assigned all the responsibility for the experimental inefficiencies of OER and ORR electrocatalysts. This triggered a research paradigm based on breaking such scaling relation that still lasts until this day (see the diagram next to this text). After noting in 2018 that breaking the scaling relation between *OOH and *OH does not necessarily entail an improvement of the OER overpotential, my group moved away from the mainstream and has since been devising alternative descriptors and methods to enhance OER electrocatalysts and bifunctional OER/ORR electrocatalysts.

In this Account, I will describe when and why we introduced the concepts of electrochemical symmetry, delta-epsilon optimization, bifunctional volcano plot, and error awareness, among others, aiming to provide quantitative tools for the computational design and optimization of electrocatalysts.

## Graphical abstract

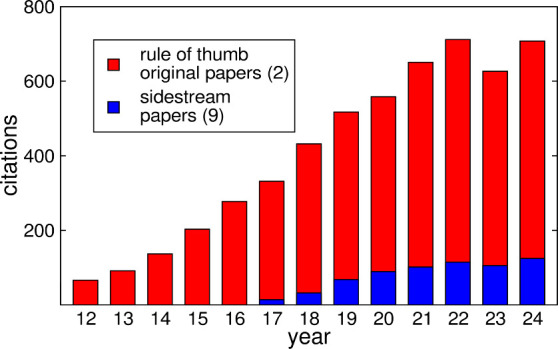

-

Govindarajan, N. ; García-Lastra, J. M. ; Meijer, E. J. ; Calle-Vallejo, F. Does the breaking of adsorption-energy scaling relations guarantee enhanced electrocatalysis? Curr. Opin. Electrochem. 2018, 8, 110–117.^1^ We showed for the first time that there is no correlation between OER overpotential and the breaking of the *OOH vs *OH scaling relation. We proposed ESSI as an alternative OER descriptor and extended it to the ORR.
-

Govindarajan, N. ; Koper, M. T. M. ; Meijer, E. J. ; Calle-Vallejo, F. Outlining the Scaling-Based and Scaling-Free Optimization of Electrocatalysts. ACS Catal. 2019, 9, 4218–4225.^2^ We devised delta-epsilon optimizations to quantitatively enhance OER electrocatalysts by following and/or breaking adsorption-energy scaling relations. Delta-epsilon optimizations can be applied to individual materials or in a high throughput manner.
-

Romeo, E. ; Illas, F. ; Calle-Vallejo, F. A general but still unknown characteristic of active oxygen evolution electrocatalysts. Chem. Sci. 2023, 14, 3622–3629.37006685 10.1039/d2sc06832jPMC10056041 ^3^ The ESSI descriptor considers the number of electrochemical steps above 1.23 eV (*n*). We noticed that OER catalysts with *n* = 3 are highly active, close to the volcano top and electrochemically symmetric.
-

Sargeant, E. ; Illas, F. ; Rodríguez, P. ; Calle-Vallejo, F. On the shifting peak of volcano plots for oxygen reduction and evolution. Electrochim. Acta 2022, 426, 140799.^4^ We derived formulas to quantify the impact DFT errors in O_2_ have on the OER and ORR volcano plots and the OER/ORR bifunctional index. The volcano peaks shift vertically for the OER and diagonally for the ORR.

## 1 When Things Got Simple

I often think about Einstein’s quote: “everything should be made as simple as possible, but not simpler”. This qualitative statement tells me that there is a point when a simple model becomes oversimplified. If simplicity is good but oversimplicity is not, where is the dividing line? The answer likely changes drastically from one scientist to the next, but I think we cross the line when the model is not sufficiently predictive. Note in passing that Einstein did not formulate his idea in those exact terms, and the sentence is presumably a simplification of an actual, denser phrase.^5^

Consider the oxygen evolution reaction (OER) in acid: 2H_2_O → *O*_2_ + 4H^+^ + 4e^–^. This is an intricate process. Probably one of the few consensuses we have in the field is that precisely its intricacies make it largely inefficient, the problem being that efficient OER electrocatalysis is needed for water (and CO_2_) electrolyzers, regenerative fuel cells, and some metal-air batteries to become daily use technologies.^6–9^ To tackle the OER from a computational perspective, numerous assumptions are needed. In this Account, I will give my view on the point when things were simple and got simpler. This article is not an up-to-date summary of the incredibly vast state of the art. Rather, it is a collection of highlights from my research, initially on the OER mainstream and later on a sidestream.

First of all, there are numerous OER pathways^9^ and we must choose one, although recent works analyze several competing pathways.^10^ Solvent, electrolyte, and double-layer effects together with pH, kinetics, mass transport effects are amply discussed elsewhere.^9^ The most widely used pathway in the recent literature is^9,11–13^

*+H2O→OH*+H++e− 1

OH*→O*+H++e− 2

O*+H2O→OOH*+H++e− 3

OOH*→*+O2+H++e− 4

There are three adsorbed intermediates in eqs 1–4: *O, *OH, and *OOH, see Figure 1. The reaction free energies of eqs 1–4 can be written in terms of the adsorption energies of those three intermediates, see eqs 5–8. Note that the adsorption energies are defined with respect to water and proton–electron pairs, which are approximated to hydrogen via the computational hydrogen electrode (CHE).^14^

$$\Delta G_{1}=\Delta G_{OH}$$ 5

$$\Delta G_{2}=\Delta G_{O}-\Delta G_{OH}$$ 6

$$\Delta G_{3}=\Delta G_{OOH}-\Delta G_{O}$$ 7

$$\Delta G_{4}=\Delta G_{O_{2}}-\Delta G_{OOH}$$ 8

where Δ*G*_O_2__ is the free energy of O_2_ on a thermodynamic scale in which the free energies of formation of water and proton–electron pairs are zero. In such a scale, Δ*G*_O_2__ = 4.91 eV in experiments (I recently noticed that Δ*G*_O_2__ is 4.91 eV, not 4.92 eV, which is the value generally used in the literature).

**Figure 1:**
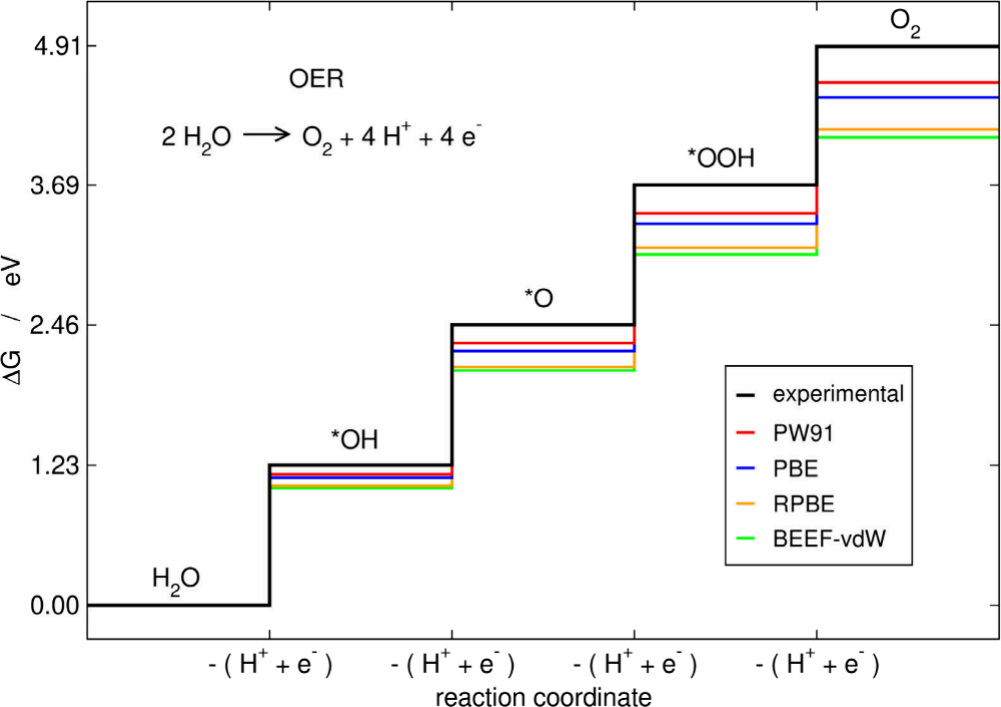
Free-energy profile of the ideal OER catalyst calculated with experimental data and some common exchange-correlation functionals. This catalyst has Δ*G*_1_ = Δ*G*_2_ = Δ*G*_3_ = Δ*G*_4_ = Δ*G*_O_2__/4. DFT errors in O_2_ cause cumulative deviations at every electrochemical step. Adapted from ref 15. Available under a CC BY 4.0 license. Copyright 2021, Sargeant et al.

The sum of eqs 5–8 is Δ*G*_O_2__, so that the experimental OER equilibrium potential is *U* ^0^ = 1.23 V_RHE_, as four electrons are transferred in every OER cycle. However, this is just the experimental value and density functional theory (DFT) calculations often give predictions far from it^4,15^ because the triplet state of O_2_ is miscalculated by nearly all common DFT exchange-correlation functionals.^16^ Here the modeling becomes semiempirical, as Δ*G*_O_2__ is also assumed to be 4.91 eV regardless of the functional used in the calculations. Indeed, Figure 1 shows the total errors in O_2_ and the cumulative errors in the free-energy profile of the ideal OER catalyst for common DFT functionals.^15^

The ideal catalyst is a hypothetical material that sets a limit for electrocatalytic efficiency without violating the first law of thermodynamics. It has all steps in a given electrocatalytic pathway of identical size, and the magnitude of each step is numerically equal to the equilibrium potential. Specifically, the ideal OER catalyst has Δ*G*_1_ = Δ*G*_2_ = Δ*G*_3_ = Δ*G*_4_ = 1.23 eV at 0 V_RHE_ (equivalent to Δ*G*_*i*_ = 0 at 1.23 V_RHE_), such that η_OER_ = 0 V.^12,15^ Thermodynamic overpotential (η_OER_) is a simple metric defined to anticipate the OER electrocatalytic activity of materials:^11,12^

$$\eta_{OER}=\Delta G_{\max}/e^{-}-U^{0}=\max\left(\Delta G_{1},\Delta G_{2},\Delta G_{3},\Delta G_{4}\right)/e^{-}-U^{0}$$ 9

where *e* ^–^ is the charge of the electron (1 if Δ*G*_*i*_ is in eV), and the step corresponding to Δ*G*_max_ is the potential-limiting step (PLS). There is a conceptual difference between PLS and rate-determining step,^17,18^ but they are often assumed to coincide.

Linear scaling relations between Δ*G*_O_, Δ*G*_OH_, and Δ*G*_OOH_ for various materials in the way shown in the insets of Figure 2 were noticed in early works.^11,19^ However, it was not until 2011 that two papers emphasized the alleged negative effect of those relations on η_OER_.^12,13^ To illustrate this, let us sum eqs 2 and 3 and eqs 6 and 7:

OH*+H2O→OOH*+2H++2e− 10

$$\Delta G_{2+3}=\Delta G_{OOH}-\Delta G_{OH}$$ 11

**Figure 2:**
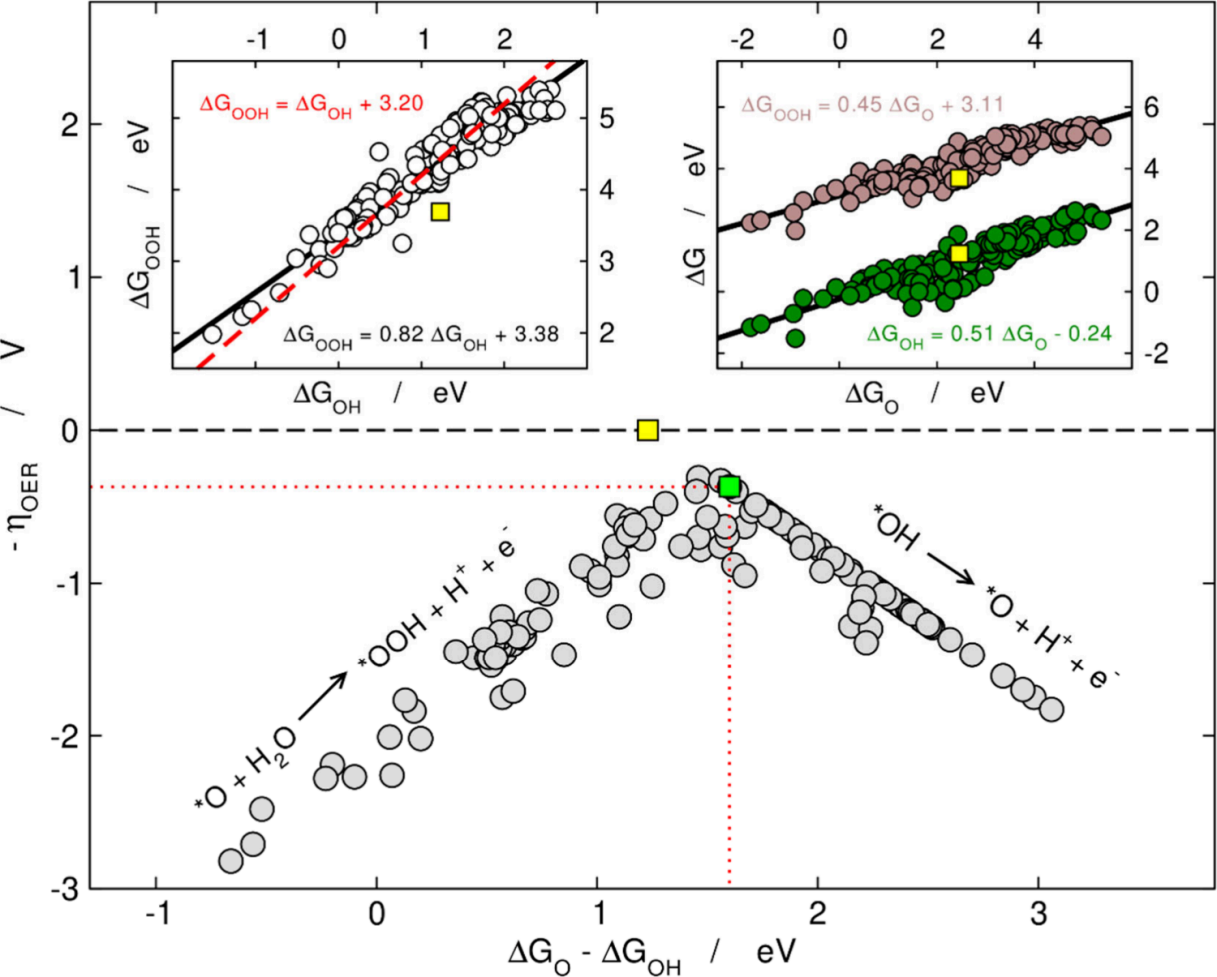
Volcano plot for η_OER_ as a function of Δ*G*_O_–Δ*G*_OH_. Left inset: scaling relation between Δ*G*_OOH_ and Δ*G*_OH_. The red dashed line is from ref 12. Right inset: scaling relation between the adsorption energies of *OOH, *OH, and *O. The least-squares linear fits are provided in each case in black together with the respective equations. The ideal/optimal OER catalyst is shown in yellow/green. Adapted from ref 1. Copyright 2018, Elsevier. Adapted from ref 20. Copyright 2020, Royal Society of Chemistry (RSC).

Those two works observed that the ideal catalyst displays Δ*G*_2 + 3_^*ideal*^ = 1.23 + 1.23 eV = 2.46 eV, while real catalysts display Δ*G*_2 + 3_^*real*^ ≈ 3.20 eV (Figure 2, left inset).^12,13^ This causes the maximal activity attainable by real catalysts to be far from that of the ideal catalyst, as seen in Figure 2. Statistically speaking, the most common PLSs are eqs 2 and 3 (Figure 4, inset).^15^ Therefore, the least overpotential is found when Δ*G*_2_ = Δ*G*_3_. When such a condition is fulfilled, combining eqs 6 and 7 we have

$${\left(\Delta G_{O}-\Delta G_{OH}\right)}^{\mathrm{optimal}}=\frac{\Delta G_{OOH}-\Delta G_{OH}}{2}\approx \frac{3.20\;eV}{2}=1.60\;eV$$ 12

According to eq 9 the least overpotential is η_OER_^optimal^ = 1.60–1.23 V = 0.37 V.^12,13^ The widespread volcano plot in Figure 2 correlating Δ*G*_O_–Δ*G*_OH_ and −η_OER_ has the ideal catalyst at (1.23 eV, 0 V) while the volcano apex is at (1.60 eV, −0.37 V) as a result of the suboptimal energetic relationship between *OOH and *OH.

## 2 When Things Got Simpler

After noting that Δ*G*_OOH_ and Δ*G*_OH_ set the volcano summit far from the ideal catalyst, lowering their difference from 3.20 to 2.46 eV to boost OER electrocatalysis became an active area of research. “Breaking scaling relations” is now a common phrase in papers and conference presentations^9,21,22^ and the concept was extended to reactions such as CO_2_ electroreduction.^23–25^ The slow pace of improvement in the OER and ORR fields was assigned to this scaling relation.^22^ Various papers schematized how to break^26–28^ or circumvent it.^29^ In sum, we got ourselves a simple, convenient and supposedly universal rule of thumb: break the *OOH vs *OH scaling relation to accelerate the OER. The net result was that we stopped looking for better catalysts and started looking just for those breaking that scaling relation. In hindsight, it is astonishing that nobody (including myself) verified quantitatively whether that hypothesis would truly enhance OER electrocatalysis for so many years.

In 2017, while working on *O, *OH and *OOH solvation on metalloporphyrins, we realized there had to be more to it than we initially thought, as scaling relations were broken when the DFT calculations were made in vacuum but were restored when the solvent was implicitly included,^30^ see Figure 3. This was an interesting finding, but it was disappointing that for no catalyst did we observe that breaking the *OOH vs *OH scaling relation improved η_OER_ below 0.37 V. Besides, a similar phenomenon had been reported earlier for hangman porphyrins.^31^ A year later, I was invited to prepare a review article in which we plotted for the first time η_OER_ as a function of the degree of breaking of the *OOH vs *OH scaling relation $\left(\gamma_{OOH/OH}=\frac{\Delta G_{OOH}-\Delta G_{OH}-2.46}{2e^{-}}\right)$ and found no correlation whatsoever,^1^ see Figure 4.

**Figure 3:**
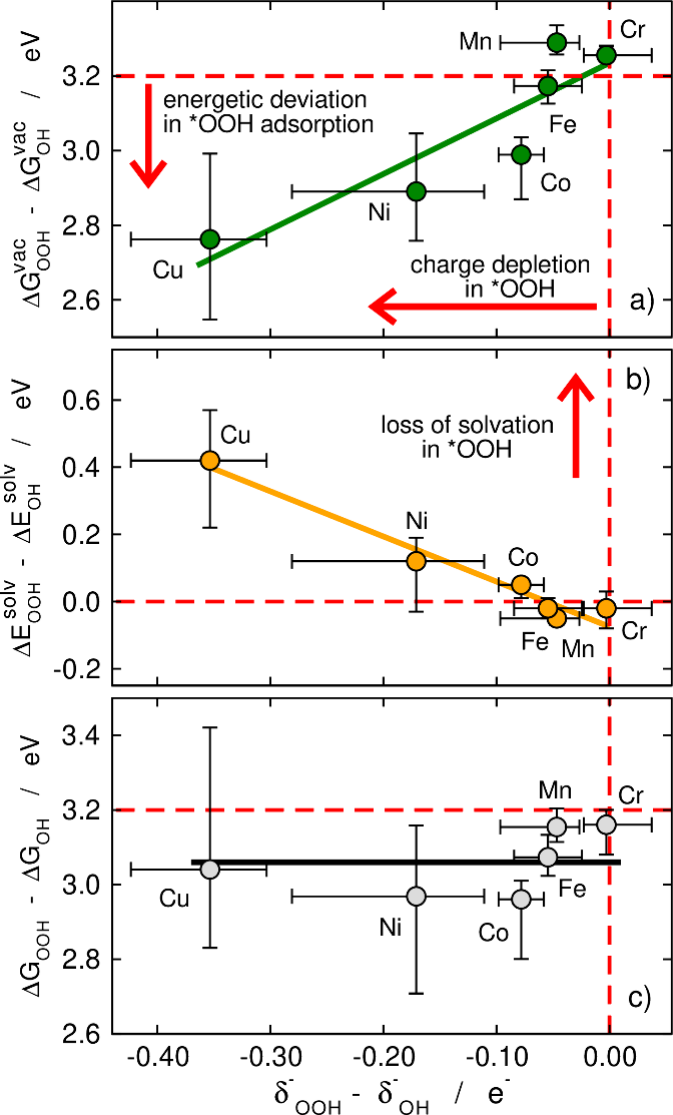
Why scaling relations are broken in vacuum and restored in solution. Differences in (a) adsorption energies of *OOH and *OH in vacuum, (b) solvation energies of *OOH and *OH, and (c) *OOH and *OH adsorption energies including solvation energies as a function of the difference in electronic charges for *OOH and *OH on metalloporphyrins of transition metals (different ring ligands lead to the error bars). In (a) and (b) the deviations from ideality grow in opposite directions, so that the trends in (c) are nearly flat. The red dashed lines show a regular situation with *OOH and *OH having identical charges and solvation energies and their energy difference being 3.20 eV. Adapted from ref 30. Available under a CC BY-NC 3.0 license. Copyright 2017, Calle-Vallejo et al.

**Figure 4:**
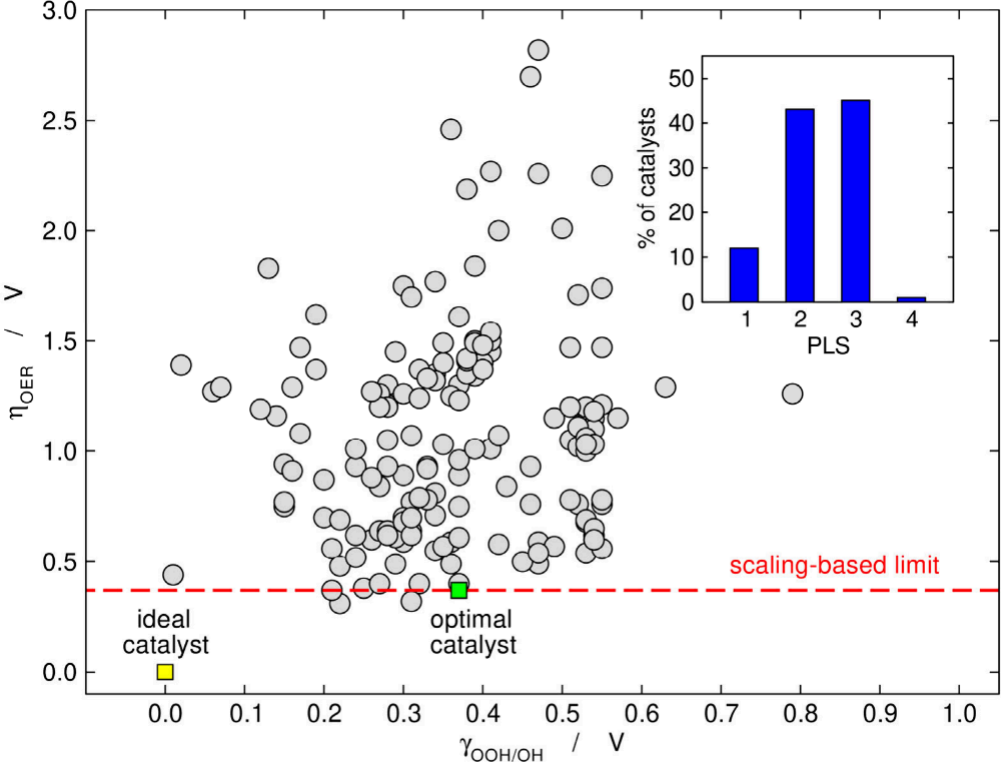
η_OER_ as a function of the degree of breaking of the scaling relation between Δ*G*_OOH_ and Δ*G*_OH_. The ideal and optimal catalysts are shown for comparison. The red dashed line is η_OER_ = 0.37 V. Inset: percentage of OER catalysts limited by eqs 1–4. Adapted from ref 1. Copyright 2018, Elsevier.

Why does the rule of thumb fail? First, when one focuses exclusively on γ_*OOH*/*OH*_, one optimizes the sum of two electrochemical steps (eqs 10 and 11) without looking at the actual PLS. Stabilizing *OOH is only a good recipe for materials the PLS of which is eq 3 (*O + H_2_O → *OOH + H^+^ + e^–^), and only around 45% of materials belong to this group, see the inset of Figure 4.^1^ There is no effect on materials limited by the eqs 1 and 2 (* + H_2_O → *OH + H^+^ + *e* ^–^, *OH → *O + H^+^ + e^–^) because *OOH is not involved in those. Around 12 and 43% of materials are respectively limited by those two steps. Finally, materials limited by step 4 (*OOH → * + O_2_ + H^+^ + e^–^) deserve special attention, as stabilizing *OOH on those actually increases η_OER_. Although only around 1% of materials are limited by step 4, the reverse of this step usually limits the oxygen reduction reaction on weak-binding materials.^1^ Note in passing that more sophisticated analyses highlighted the importance of stabilizing the transition state leading to *OOH^32^ and the kinetics of *OOH dehydrogenation, the O–O bond-making step, and the final desorption of O_2_.^33,34^

In brief, the *OOH vs *OH rule of thumb has no effect on 55% of the materials or may even increase the overpotential in some cases.^1,15^ Hence, one should probably focus on the PLS of each material instead of stabilizing *OOH by default on all materials and at all costs.

The lack of correlation between γ_OOH/OH_ and η_OER_ indicates that it is unnecessary to stabilize Δ*G*_OOH_ in every case. Instead, we should make real catalysts approach the ideal one. To quantify the similarity between real and ideal catalysts, we needed a new descriptor incorporating information from various electrochemical steps, not just the sum of steps 2 and 3. We ended up devising the electrochemical-step symmetry index (ESSI):^1^

$$ESSI=\frac{1}{n}\overset{n}{\underset{i=1}{\sum }}\left(\frac{{\Delta G}_{i}^{+}}{e^{-}}-U^{0}\right)$$ 13

where Δ*G*_*i*_^+^ are the free energies of eqs 5–8 equal to or larger than *e* ^–^ *U* ^0^ = 1.23 eV, and *n* is the count of those steps. Because of the first law of thermodynamics, no catalyst displays *n* = 0, and the ideal catalyst has *n* = 4 and ESSI = 0. In turn, real catalysts are found in between: 1 ≤ *n* ≤ 3. As shown in Figure 5, η_OER_ generally decreases alongside ESSI. Besides, by definition: ESSI ≤ η_OER_.^1,20^

**Figure 5:**
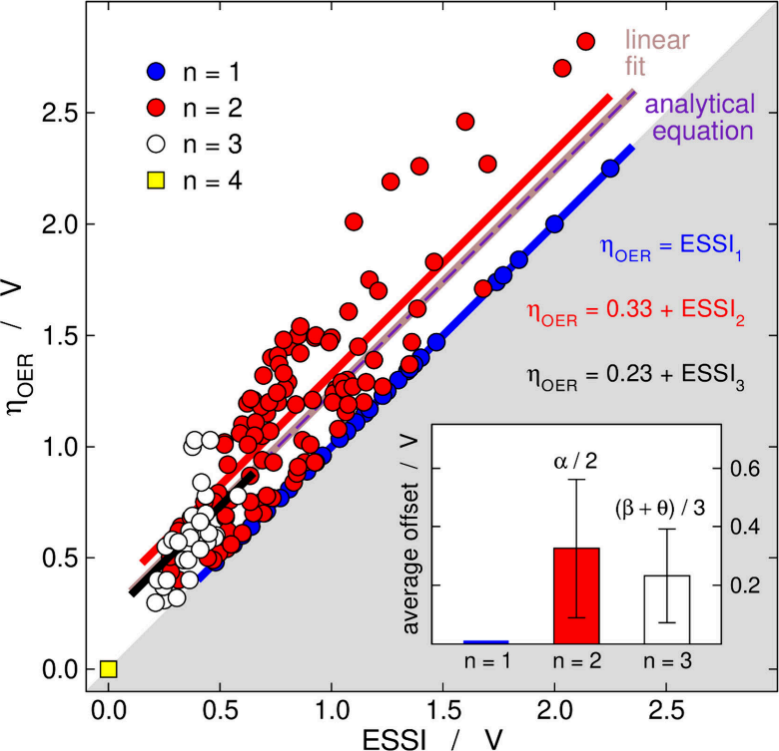
η_OER_ as a function of ESSI. High OER activity should be observed when ESSI approaches zero. There are no materials in the gray zone. The ideal catalyst is shown for comparison. The linear fit to the data is η_OER_ = 1.00 ESSI + 0.25, and the analytical equation is $\eta_{OER}\approx ESSI+w_{2}\cdot avg\left(\frac{\alpha }{2}\right)+w_{3}\cdot avg\left(\frac{\beta +\theta }{3}\right)$ , with *w*_2_ = 0.625 and *w*_3_ = 0.179. Inset: average values of $\frac{\alpha }{2}$ and $\frac{\beta +\theta }{3}$ (0.33 and 0.23 V, respectively). Hence, the analytical equation is η_OER_ ≈ ESSI + 0.25. Adapted from ref 36. Copyright 2025, American Chemical Society (ACS).

For example, consider the double perovskite Sr_2_NiIrO_6_,^35^ which has: Δ*G*_OH_ = 0.77 eV, Δ*G*_*O*_ = 2.12 eV and Δ*G*_OOH_ = 3.25 eV. The energies of the OER steps are Δ*G*_1_ = 0.77 eV, Δ*G*_2_ = 1.35 eV, Δ*G*_3_ = 1.13 eV, Δ*G*_4_ = 1.67 eV. The number of steps over 1.23 eV is *n* = 2 (steps 2 and 4), and the PLS is step 4. Although the *OOH vs *OH scaling relation is broken (γ_OOH/OH_ = 0.01 V), the overpotential is η_OER_ = 0.44 V, and ESSI = 0.28 V. Sr_2_NiIrO_6_ is only one of many examples of materials totally or partially breaking the *OOH vs *OH scaling relation but displaying η_OER_ > 0.37 V (Figure 4).^1,36^ We learned from all this that the ideal catalyst breaks the *OOH vs *OH scaling relation, but that does not necessarily mean that when a real catalyst breaks it the overpotential will decrease.

I stress here that nowadays most descriptors in electrocatalysis are ad hoc. They may have a visible correlation within a specific data range with an activity metric, but the physics or chemistry underneath are often unknown, handwaving or nonexistent. This is not the case of ESSI, as it has a cause-effect relationship with η_OER_ and it is possible to determine the parameters of the correlation.^36^ I will skip the derivation here and say that the linear fit in Figure 5 is η_OER_ = 1.00 ESSI + 0.25, and the analytical prediction is precisely η_OER_ ≈ ESSI + 0.25.^36^ Besides, ESSI can be evaluated irrespective of scaling relations between OER intermediates and the specific choice of reaction pathway.

## 3 Simple and Quantitative Alternatives

After realizing that breaking the *OOH vs *OH scaling relation is not a univocal design guideline, we started thinking about truly quantitative ways of optimizing OER materials. As ESSI is an average, it features range bars showing the dispersion of the data. We thought catalysts with wide bars should be easier to optimize than those with narrow bars because one can more easily lower the PLS until another step becomes limiting.^1^

With this in mind, we devised a scaling-free procedure and a scaling-based optimization procedure. The two methods can be applied separately, in series or simultaneously, and we called the collection of all possible options δ−*ε* optimization.^2^ Our rationale was that one can decrease η_OER_ by following scaling relations and/or by breaking them. When lowering η_OER_ following scaling relations, if a given material *i* experiences a shift in Δ*G*_OH_ by as much as δ, the slopes of scaling relations ensure that Δ*G*_OOH_ should approximately be shifted by δ, and Δ*G*_*O*_ by 2δ. Hence, a new Δ*G*_OH,*i*_′ = Δ*G*_OH,*i*_ + δ, is accompanied by Δ*G*_OOH,*i*_′ = Δ*G*_OOH,*i*_ + δ, and Δ*G*_O,*i*_′ = Δ*G*_O,*i*_ + 2δ. In turn, to lower η_OER_ breaking the *OOH vs *OH scaling relation we introduced a shift *ε* in Δ*G*_OOH_ that has no effect on Δ*G*_O_ and Δ*G*_OH_ (Δ*G*_OOH_″ = Δ*G*_OOH_ + *ε*). These simple considerations turn eqs 5–8 into [Disp-formula eq14]-[Disp-formula eq17].^2^

$$\Delta G_{1}=\Delta G_{OH}+\delta$$ 14

$$\Delta G_{2}=\Delta G_{O}-\Delta G_{OH}+\delta$$ 15

$$\Delta G_{3}=\Delta G_{OOH}-\Delta G_{O}-\delta +\varepsilon$$ 16

$$\Delta G_{4}=\Delta G_{O_{2}}-\Delta G_{OOH}-\delta -\varepsilon$$ 17

As required by the first law of thermodynamics, the sum of eqs 14–17 is still Δ*G*_O_2__ = 4.91 eV. With these equations, one can run an automated minimization of η_OER_ as a function of the free parameters δ and ε, see Figure 6. δ > 0 implies a weakening of the adsorption energies, while δ < 0 implies their strengthening. As *ε* is a stabilization of *OOH, it is always negative. Consider again Sr_2_NiIrO_6_:^35^ δ = 0.16 eV weakens the adsorption energies: Δ*G*_OH_ = 0.93 eV, Δ*G*_O_ = 2.44 eV and Δ*G*_OOH_ = 3.41 eV. The OER steps are now: Δ*G*_1_ = 0.93 eV, Δ*G*_2_ = 1.51 eV, Δ*G*_3_ = 0.97 eV, Δ*G*_4_ = 1.51 eV. In turn, η_OER_ is lowered from 0.44 to 0.28 V, and the PLSs are steps 2 and 4.

**Figure 6:**
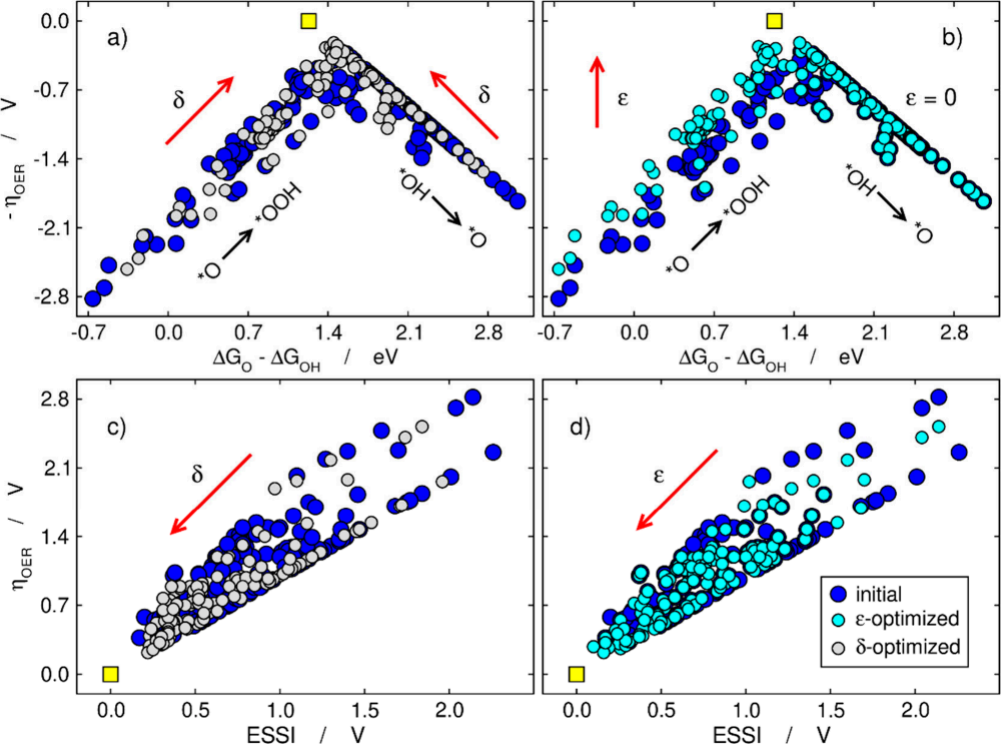
Effects of (a) δ optimization and (b) ε optimization on the OER volcano. δ optimizes catalysts on both sides of the volcano, and ε optimizes only those on the left side. Effects of (c) δ optimization and (d) ε optimization on the relationship between η_OER_ and ESSI. The ideal catalyst is shown in yellow. Adapted from ref 2. Copyright 2019, ACS.

Originally, we suggested that conservative ranges for δ and ε were [−0.3, 0.3] and [−0.3, 0] eV, respectively. These ranges guarantee that the active sites are modified but do not lose their initial identity.^2^ For instance, strained Pt(111) in the range of [−3%, 3%] does not experience important geometric changes other than the contraction or elongation of Pt–Pt bonds, while Δ*G*_OH_ undergoes respective changes in the range of [0.15, −0.15] eV with respect to unstrained Pt(111) (see Figure 11, inset).^2,37,38^ Apart from strain, δ may also correspond to moderate electronic or (second-shell) coordination effects.^38–41^ In turn, *ε* may be a stabilization of *OOH via interactions (such as hydrogen bonding) with ligands or functional groups near the active site, and by seizing nanoscale confinement or ensemble effects.^2,27,28,31,42,43^ It is clear in Figure 6a,b that δ optimization displaces most data points on both sides of the volcano toward the apex while *ε* does it only on the left side. In turn, Figure 6c,d show that η_OER_ decreases alongside ESSI, so that more electrochemical symmetry generally means lower overpotential. Finally, other scaling relations can also be broken. For example, if a catalyst is limited by eq 2, suitably breaking the scaling relation between *O and *OH might enhance the OER activity.^44,45^

## 4 Extensions and Simplifications along the Sidestream

Since each electrocatalytic reaction has an equilibrium potential, one can always establish the free-energy diagram of the ideal catalyst and define ESSI. To illustrate this, we extended ESSI to the ORR (in acid: O_2_ + 4H^+^ + 4e^–^ → 2H_2_O),^1^ which is often thought to follow the reverse OER pathway: step 1′, O_2_ + * + H^+^ + e^–^ → *OOH; step 2′, *OOH + H^+^ + e^–^ → *O + H_2_O; step 3′, *O + H^+^ + e^–^ → *OH; step 4′, *OH + H^+^ + e^–^ → * + H_2_O. Similarly to the OER volcano summit, that of the ORR is also shifted with respect to the ideal catalyst by 0.37 (e)­V. In this case, the most common energetic descriptor is Δ*G*_OH_ and the coordinates of the top are (Δ*G*_OH_, −η_ORR_) = (0.86 eV, −0.37 V) (see also Figure 11).^4,28^

Assuming inverse OER/ORR pathways is yet another common approximation but one that provides some insight: if one of the pathways is the reverse of the other, then ESSI_ORR_ can be rapidly calculated as a function of ESSI_OER_ and vice versa:^1^

$$ESSI_{ORR}=-\frac{n\cdot ESSI_{OER}}{4-n}$$ 18

Equation 18 shows that *n* is not only important for ESSI_OER_ but also for ESSI_*ORR*_, see Figure 7. This motivated us to make the usual volcano plot classifying the data points not in materials families but according to *n*, as shown in Figure 8a.^3^ This made us realize that materials with *n* = 3 are generally highly active for the OER, often located around the volcano apex, and highly symmetric in electrochemical terms, as shown in Figure 8c.

**Figure 7:**
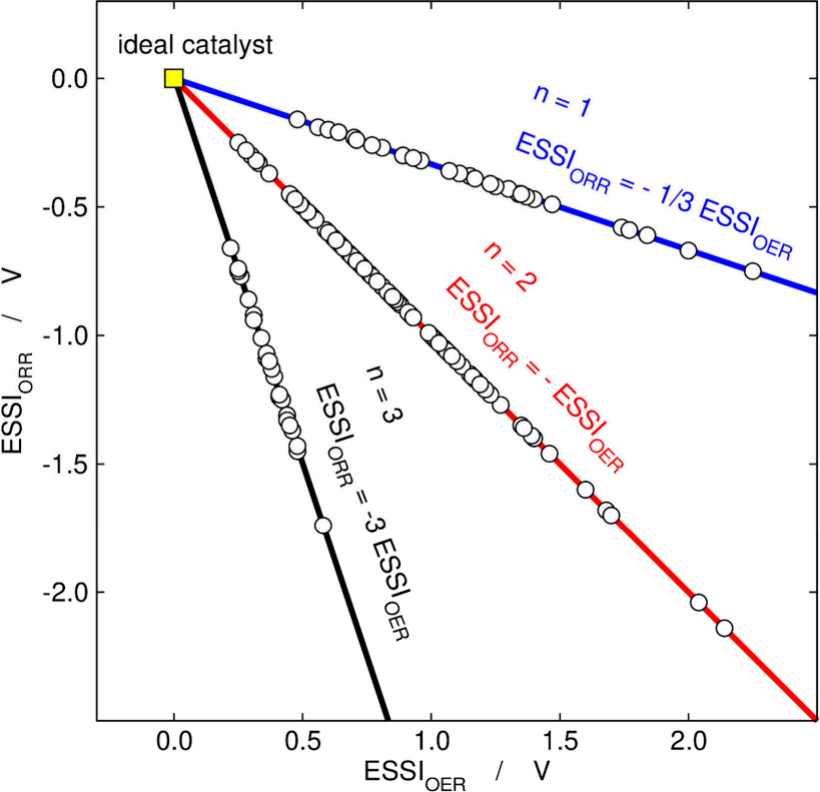
ESSI_ORR_ as a function of ESSI_OER_, calculated using eq 18. The slopes of the lines depend on the number of OER steps above 1.23 eV (*n*). Adapted from ref 1. Copyright 2018, Elsevier.

**Figure 8:**
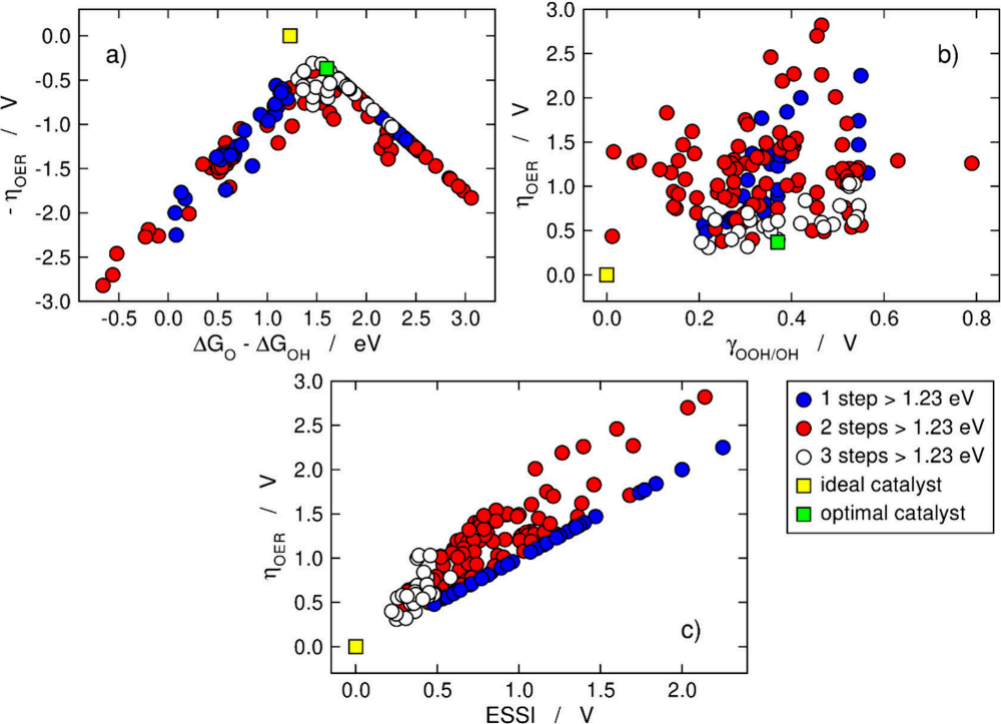
OER activity trends from Figures 2, 4, and 5 but classifying the data depending on the number of steps larger than 1.23 eV (*n*). (a) Volcano plot. (b) Overpotential versus γ_OOH/OH_. (c) Overpotential versus ESSI. The ideal/optimal catalyst is shown in yellow/green. Adapted from ref 3. Available under a CC BY 3.0 license. Copyright 2023, Romeo et al.

With ESSI_OER_, ESSI_ORR_, η_OER_ and η_ORR_ we were able to make a simple analysis of OER/ORR bifunctionality. This is important for some metal-air batteries and regenerative fuel cells, where electrocatalysts should be active for both reactions.^6,7^ Here, an intuitive and useful metric is the bifunctional index (BI),^46–48^ experimentally defined as the difference between the potentials needed to achieve an OER current density of 10 mA/cm^2^ and an ORR current density of −1 mA/cm^2^: BI_exp_ = *U*_OER@10 mA/cm^2^_–*U*_ORR@-1 mA/cm^2^_. In turn, the computational bifunctionality index is defined as^48^

$$BI_{DFT}=U_{OER}^{L}-U_{ORR}^{L}=\eta_{OER}+\eta_{ORR}$$ 19

where *U*_OER_^L^ = Δ*G*_max_/*e* ^–^ and *U*_ORR_^L^ = −Δ*G*_max_′/*e* ^–^. The ideal OER/ORR bifunctional catalyst has BI_DFT_ = 0, while the optimal OER/ORR catalyst has sites located at the top of both volcanoes, such that BI_DFT_^optimal^ ≈ 0.37 + 0.37 V = 0.74 V. In practice, electrocatalysts generally display BIs above 1.0 V and those in the range of 0.8–0.9 V are considered highly bifunctional.^46–48^ This is shown in Figure 9 together with an inset in which there is a correlation between BI_DFT_ and the OER/ORR electrochemical symmetry described as ΔESSI = ESSI_OER_–ESSI_ORR_.^48^ Some authors claim that one can go below BI_DFT_^optimal^ ≈ 0.74 V (ref 49 and references therein), which is also possible in our approach, as the limit considering error bars is 0.74 ± 0.20 V. Hence, the hard limit without a significant destabilization of scaling relations is BI ≥ 0.54 V.

**Figure 9:**
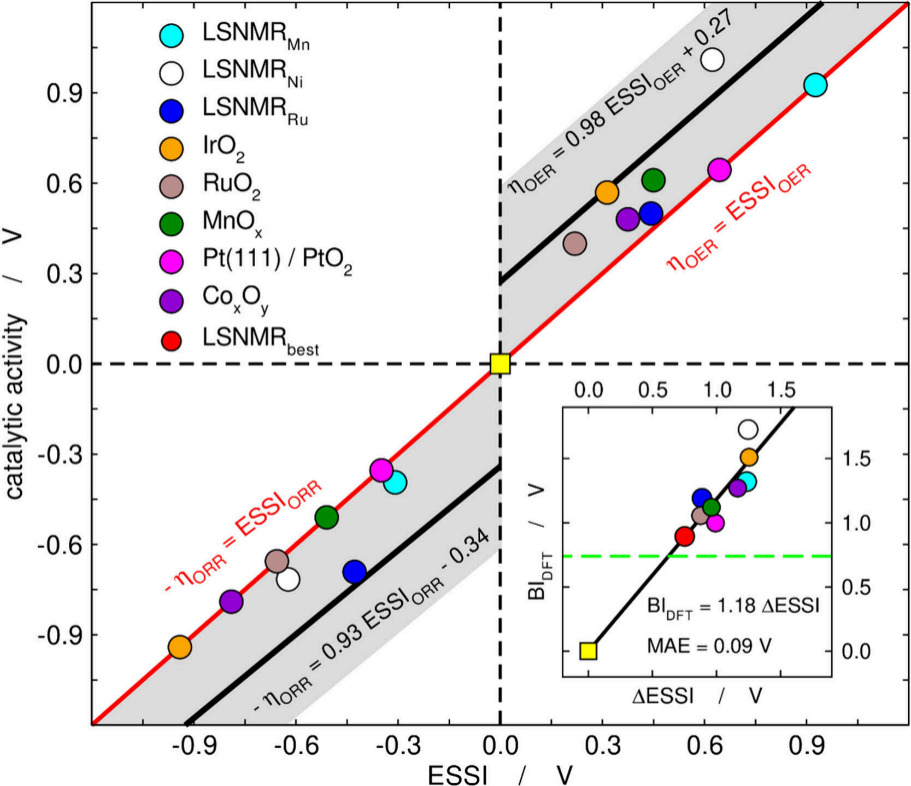
OER/ORR catalytic activity of various oxides as a function of ESSI. For the OER, the catalytic activity is η_OER_, while it is −η_ORR_ for the ORR. The vertical differences between the corresponding OER/ORR points are the *BIs* of materials. The gray area spans a confidence interval of ∼85% (approximately ±0.3 eV around the black lines). Inset: correlation between BI_DFT_ and ΔESSI = ESSI_OER_–ESSI_ORR_. LSNMR: La_1.5_Sr_0.5_NiMn_0.5_Ru_0.5_O_6_. The subindices of LSNMR indicate the OER/ORR active sites. LSNMR_best_ has Ru/Mn sites for the OER/ORR. Adapted from ref 48. Copyright 2019, ACS.

In 2021, I was invited to write an article on single-atom electrocatalysts (SACs).^50^ In response, we created an OER/ORR bifunctional volcano plot for SACs based on Δ*G*_O_2__ and Δ*G*_OOH_–Δ*G*_OH_ ≈ 3.20 eV.^50^ Our analysis showed that there are three regions in the bifunctional volcano (Figure 10): region I (Δ*G*_OH_ < 0.86 eV), where BI_DFT_ decreases as Δ*G*_OH_ becomes increasingly positive. Region II is a plateau in which, on average, BI_DFT_ ≈ 1.48 V for 0.86 eV < Δ*G*_OH_ < 1.60 eV. Admittedly, data are scattered in this region, but the limit (BI_DFT_^optimal^ ≈ 0.74 V) is not trespassed. Region III (Δ*G*_OH_ > 1.60 eV), where BI_DFT_ increases as a function of Δ*G*_OH_.^50^

**Figure 10:**
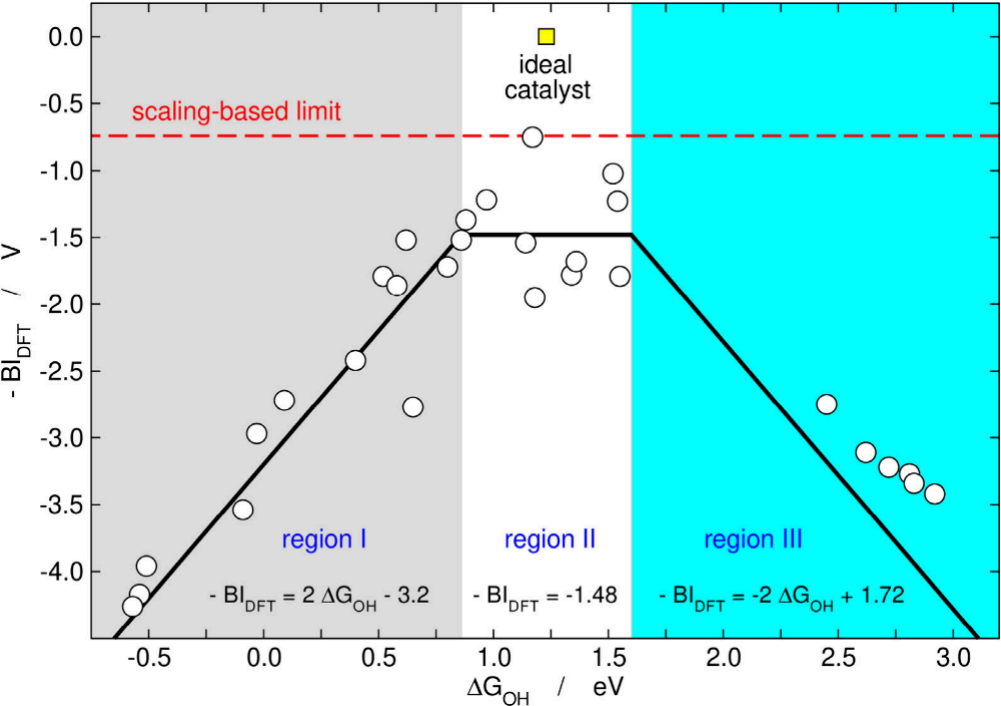
Bifunctional volcano plot for OER/ORR electrocatalysis on single-atom catalysts. There are three regions depending on Δ*G*_OH_. Adapted from ref 50. Available under a CC BY-NC 3.0 license. Copyright 2022, Kolb and Calle-Vallejo.

In sum, all this section indicates that ESSI is not limited to the OER and *n* is a simple and important parameter to classify catalysts. In particular, *n* = 3 is the value to aim for when optimizing existing OER catalysts or designing new ones.

## 5 Error Awareness

DFT errors for the thermochemistry of molecules and solids have been known for decades, are sizable, and affect numerous compounds.^16,51–57^ Currently, the computational electrocatalysis community is well aware of the error in O_2_ (*ε*_*O*_2__)^14,15^ but seems oblivious to countless other errors.^58^ What is more, the impact of DFT errors in our conclusions is still largely omitted^58–60^ and this is, in my view, among the reasons why our models have limited predictiveness.

Apart from showing the effects of ε_O_2__ in the free-energy diagram of the ideal catalyst (Figure 1), my group also analyzed the impacts on volcano plots for the OER and ORR, see Figure 11.^4^ Because the statistically most frequent PLSs are different (OER: eqs 2 and 3; ORR: the reverse of eqs 1 and 4), the effects are also different: OER volcanoes shift vertically downward depending on *ε*_*O*_2__, whereas ORR volcanoes shift downward and leftwards along a diagonal line. Hence, if *ε*_*O*_2__ is not corrected there are qualitative and quantitative problems in DFT-based electrocatalysis models.^4,15^ We have shown that these errors do not just affect the OER/ORR but virtually all electrocatalytic reactions, as exemplified for O_2_ reduction to H_2_O_2_,^61,62^ CO_2_ reduction to CO and methanol,^63^ CO reduction to acetate,^64^ electrochemical ammonia synthesis,^59^ and NO reduction to NH_3_ and hydroxylamine.^59,65^

**Figure 11:**
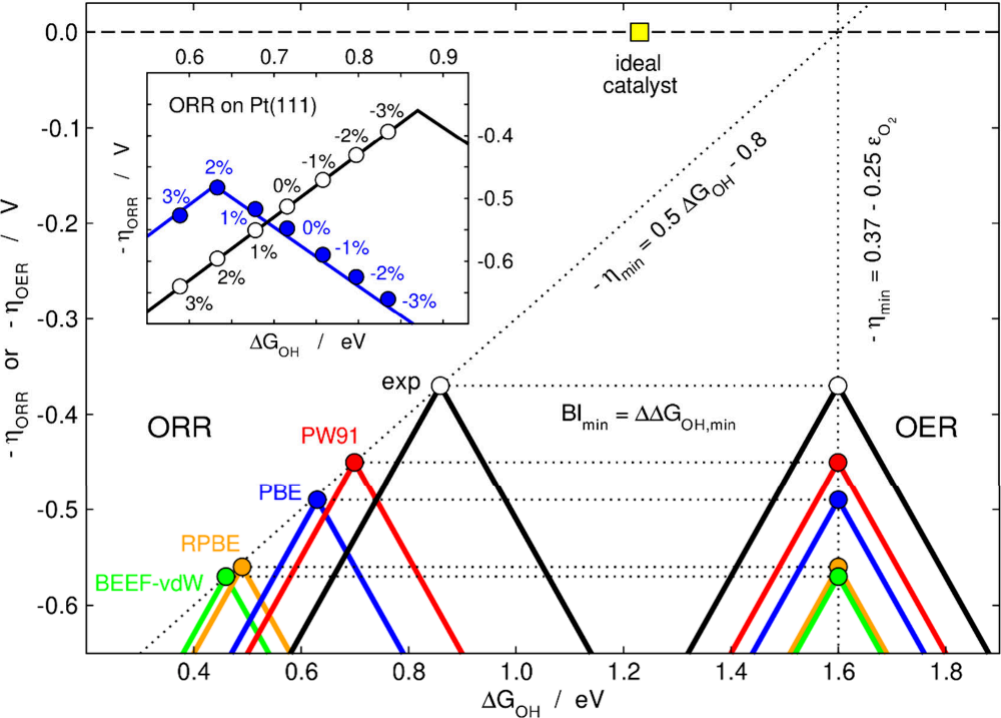
Functional-dependent OER and ORR volcano plots. Volcanoes using experimental data (exp) are also shown. The apexes shift systematically depending on *ε*_*O*_2__, which is functional specific. The apices move vertically (OER) or diagonally (ORR) because the PLSs are different. *BI*_*min*_ is given by the horizontal dashed lines connecting the volcano apices. The ideal catalyst is shown for comparison. Inset: ORR volcano plot for (un)­strained Pt(111). Negative/positive percentages indicate the compression/expansion with respect to Pt(111) (0%: unstrained Pt(111)). Adapted from ref 4. Available under a CC BY-NC-ND 4.0 license. Copyright 2022, Sargeant et al.

Therefore, the widespread notion that “the actual DFT values might be wrong but the trends are fine” is a cliché worth reconsidering. To illustrate this with a practical example, consider the ORR volcano for Pt(111) in the inset of Figure 11. Negative and positive percentages indicate compression or expansion with respect to the interatomic Pt–Pt distances in the bulk, respectively. If O_2_ is not corrected and the PBE functional is used, the blue volcano plot has a summit for Pt(111) expanded by 2%. Upon correcting the error in O_2_, the black volcano plot predicts that a compressive strain of −3% lies close to the summit. There is general agreement in the literature that Pt(111) should be compressed such that *OH adsorption weakens: Δ*G*_OH_^summit^ ≈ Δ*G*_OH_^Pt(111)^ + [0.10–0.15] eV.^40,41,66,67^

There are also DFT errors in the adsorbed state. Disturbingly, they are more difficult to detect than molecular errors and, thus, even less publicized. Such errors have a net effect on adsorption energies: for example, after it was noted that eqs 1–9 predict high OER activities for IrO_2_(110) but not for RuO_2_(110),^68,69^ we used a different kind of scaling relation to find a specific adsorption-energy error in RuO_2_(110).^70^ Usually scaling relations are established for two species adsorbed on a wide set of materials, using a single DFT code and a specific type of potentials to describe ion-electron interactions. In this case, we took a single material and adsorbed on it the two species using various codes and potentials. These scaling relations are shown in Figure 12 for Δ*G*_O_, Δ*G*_OH_ and Δ*G*_OOH_ on RuO_2_(110) and IrO_2_(110).^70^

**Figure 12:**
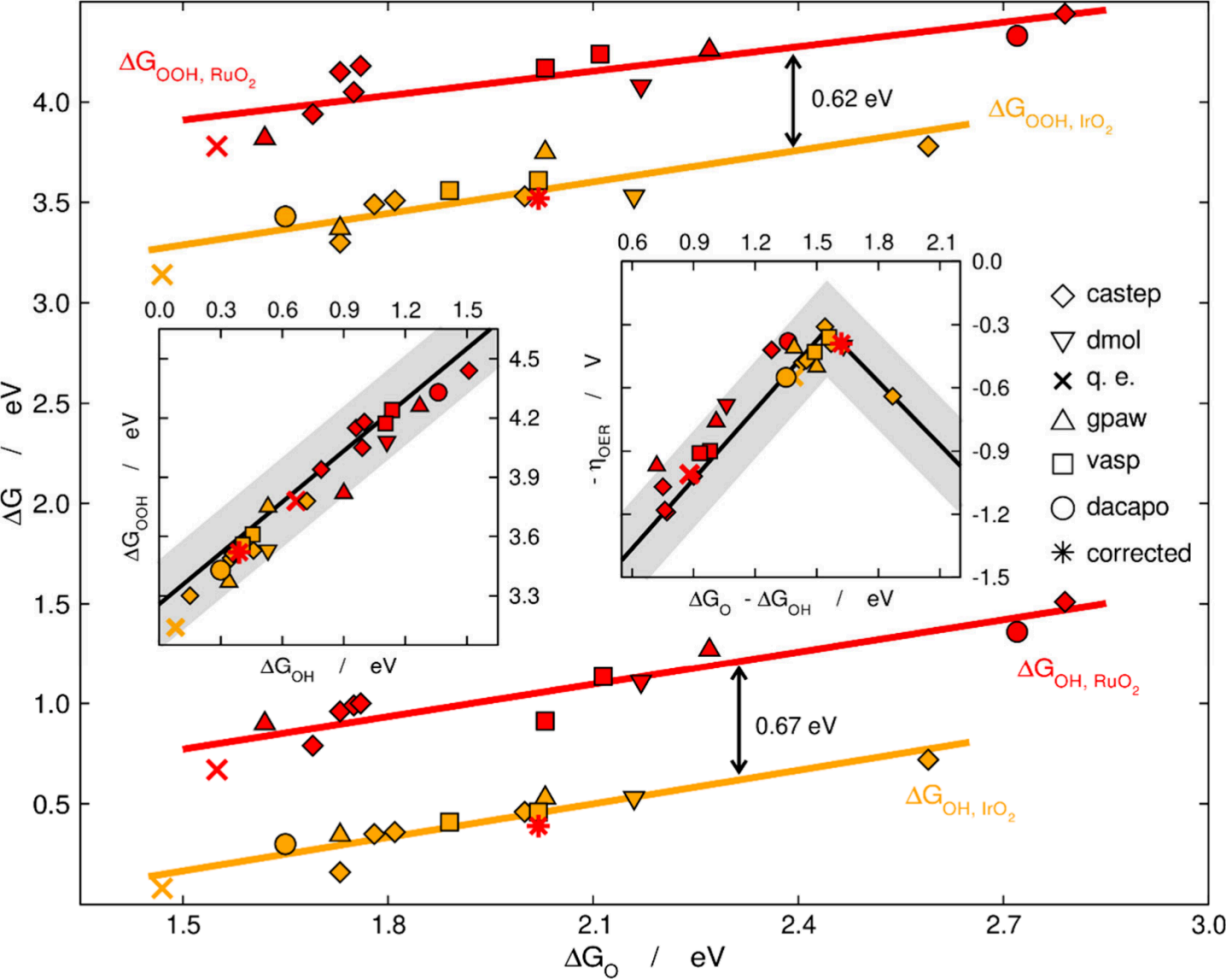
Adsorption-energy scaling relations between *OOH, *OH and *O for RuO_2_(110) and IrO_2_(110) calculated using the listed codes. q. e.: Quantum Espresso. The *O adsorption energies of RuO_2_(110) and IrO_2_(110) are similar but those of *OH/*OOH differ by 0.67/0.62 eV. Left inset: Adsorption-energy scaling relation between *OOH and *OH. Right inset: Volcano plot showing that most codes predict that IrO_2_(110) is OER active while RuO_2_(110) is inactive. The black lines are from ref 76 and the gray areas cover an area of ±0.20 eV around them. Adapted from ref 70. Copyright 2017, Wiley.

While the ranges of Δ*G*_O_ are similar for these two oxides, their Δ*G*_OH_ and Δ*G*_OOH_ differ on average by 0.67 and 0.62 eV, respectively. The left inset of Figure 12 shows that the scaling relation between *OOH and *OH is similar to that in Figure 2, such that the errors are due to either *O or simultaneously to *OH and *OOH. These nearly constant shifts are responsible for the inaccurate OER activity predictions of RuO_2_(110) in the right inset of Figure 12. However, if one shifts the average Δ*G*_OH_ and Δ*G*_OOH_ of RuO_2_(110) by 0.67 and 0.62 eV, respectively, it becomes as active as IrO_2_(110), see the asterisk in the right inset of Figure 12. Van der Waals functionals and explicit water solvation also improve the predictions.^70^ It is worth noting that other authors attribute these mispredictions to different factors,^68,69,71–73^ other approaches successfully explain the high OER activity of RuO_2_(110) and IrO_2_(110),^74,75^ and detailed works are available on the detection, mitigation and impact on catalysis of DFT errors.^58^

Finally, let me emphasize that the research efforts discussed here were made to make more accurate models. Figure 13 shows that, in general, we reach good agreement between experiments and theory for η_OER_, Δ*G*_O_–Δ*G*_OH_ and BI, and full agreement for *U* ^0^. As a result of error cancellation, the mean absolute errors (MAEs) for Δ*G*_O_–Δ*G*_OH_ and BI are lower than for η_OER_, but MAEs around 0.10 eV are satisfactory, in my opinion. Our method for assessing (Δ*G*_O_–Δ*G*_OH_)_exp_ can be used to add experimental data into DFT-based volcano plots.^35,77–79^ Briefly, the method is based on the semiempirical volcano plot for η_OER_ from experiments at 1 mA/cm_cat_^2^ as a function of Δ*G*_O_–Δ*G*_OH_ from DFT calculations by Seh et al.^22^ Knowing the experimental η_OER_, one can assess the expected Δ*G*_O_–Δ*G*_OH_. Besides, we recently detected and corrected the errors across functionals in RuO_2(s)_ and RuO_4(g)_, which opened the door for errorproof Pourbaix diagrams and akin electrochemical stability analyses.^80^ In the future, I look forward to using the descriptors and methods described here not only corroboratively but also predictively.

**Figure 13:**
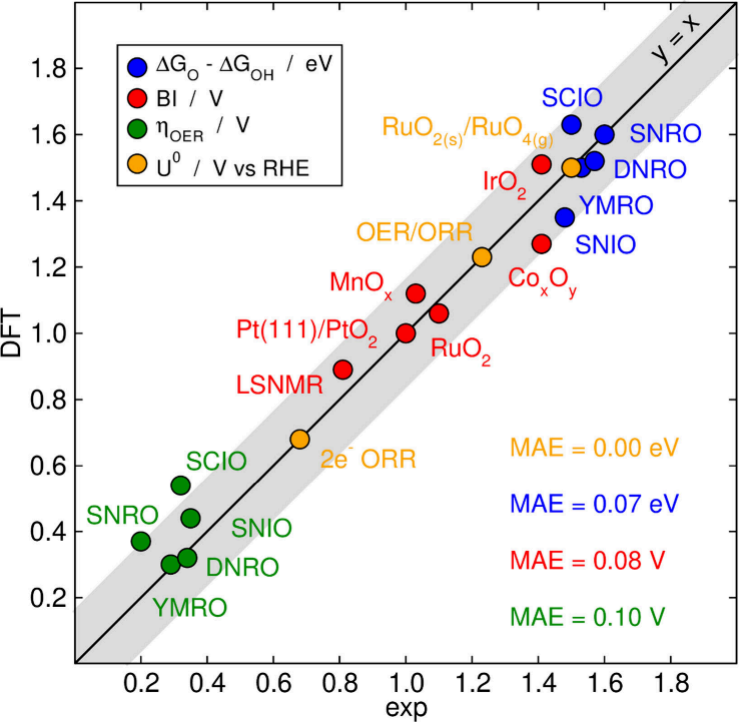
Parity plot for various parameters of interest calculated with DFT and in experiments. The mean absolute errors (MAEs) are provided in each case. The gray line covers an area of ± 0.15 (e)V around the parity line. Original figure made with data from refs 35, 48, 61, 62, and 77–80 .

## Conclusions

Honduran Guatemalan writer Augusto Monterroso penned a short story called The Dinosaur. It is only one-sentence long: “And when they woke up, the dinosaur was still there”. Looking at the figure in the Conspectus, I cannot say that the field has moved beyond the rule of thumb based on breaking the scaling relation of Δ*G*_OOH_ vs Δ*G*_OH_. Anyway, this heuristic rule motivated my transition from the OER/OER mainstream to a sidestream where my group has introduced quantitative concepts such as ESSI, γ_OOH/OH_, *n*, BI_DFT_, error awareness, and delta-epsilon optimization to model OER/ORR catalysts. Importantly, there is a mathematical causality relationship between ESSI and η_OER_, it has been shown quantitatively that delta-epsilon optimization does lower η_OER_, and analyzing the breaking of scaling relations for CO_2_ reduction, I reached similar conclusions.^25^

My group’s research suggests that one has a higher probability of enhancing a given OER catalyst by focusing on its specific potential-limiting step. We knew this before 2011 but unfortunately got distracted trying to impetuously break the *OOH vs *OH scaling relation. In addition, we found that DFT error analyses are necessary to guarantee nonincidental agreement between theory and experiments. I hope this Account helps readers rethink the present OER/ORR electrocatalyst design paradigm, so that one day the dinosaur is not there anymore.
